# Development and feasibility of an evidence-informed self-management education program in pediatric concussion rehabilitation

**DOI:** 10.1186/s12913-016-1664-3

**Published:** 2016-08-17

**Authors:** Anne W. Hunt, Luciano De Feo, Jennifer Macintyre, Dayna Greenspoon, Talia Dick, Katherine Mah, Melissa Paniccia, Christine Provvidenza, Nick Reed

**Affiliations:** 1 Bloorview Research Institute, Holland Bloorview Kids Rehabilitation Hospital, 150 Kilgour Road, Room 4W221, Toronto, Ontario M4G 1R8 Canada; 2 Department of Occupational Science & Occupational Therapy, University of Toronto, 160-500 University Ave, Toronto, Ontario M5G 1V7 Canada; 3 University of Toronto, Rehabilitation Sciences Institute, 160-500 University Ave., Toronto, Ontario M5G 1V7 Canada

**Keywords:** Education, Concussion, Children, Youth, Knowledge translation, Self-management, Pediatric

## Abstract

**Background:**

Concussion is a considerable public health problem in youth. However, identifying, understanding and implementing best evidence informed recovery guidelines may be challenging for families given the vast amount of information available in the public domains (e.g. Internet). The objective of this study was to develop, implement and evaluate the feasibility of an evidence-informed self-management education program for concussion recovery in youth.

**Methods:**

Synthesis of best evidence, principles of knowledge translation and exchange, and expert opinion were integrated within a self-management program framework to develop a comprehensive curriculum. The program was implemented and evaluated in a children’s rehabilitation hospital within a universal health care system. A retrospective secondary analysis of anonymous data from a program evaluation survey was used to evaluate program feasibility, to identify features of importance to program participants and to assess changes in participants’ knowledge.

**Results:**

The program, “Concussion & You” includes a comprehensive, evidence informed, population specific curriculum that teaches participants practical strategies for management of return to school and play, sleep, nutrition, relaxation and energy conservation. A ‘wheel of health’ is used to facilitate participants’ self-management action plan. Results from eighty-seven participant surveys indicate that the program is feasible and participant knowledge increased in all areas of the program with the highest changes reported in knowledge about sleep hygiene, rest and energy conservation.

**Conclusion:**

Findings indicate that “Concussion & You” is a feasible program that is acceptable to youth and their families, and fills a health system service gap.

**Electronic supplementary material:**

The online version of this article (doi:10.1186/s12913-016-1664-3) contains supplementary material, which is available to authorized users.

## Background

Understanding how to promote recovery in youth following concussion may be a considerable challenge for both parents and youth. Factors that contribute to this challenge include managing post-concussion symptoms, navigating return to school and sports, and developing awareness of evidence-informed policies and procedures [1]. The abundance of information in public domains (e.g., internet; social networking sites) regarding concussion management make it difficult for families to discern which approaches are valid, effective and suitable for youth [2, 3]. Self-management approaches and use of knowledge translation and exchange (KTE) principles may help promote recovery within this population [4, 5].

Mild traumatic brain injury (mTBI) or concussion is defined as “a complex pathophysiologic process affecting the brain, induced by traumatic biomechanical forces” (p.250) [6]. Concussion is a considerable public health problem in youth with a conservative estimated incidence of 754 and 440 per 100,000 for boys and girls respectively [7]. While most youth recover functionally within the first few weeks post injury, a subset (14 %) continue to report symptoms that persist beyond three months, with 2.3 % still symptomatic at one year [6]. Current best-practice guidelines recommend a period of cognitive and physical rest followed by a gradual return to school and activity [8]. However, understanding and implementing these guidelines may be challenging for families. For example, the term ‘rest’ may be difficult for families and youth to interpret. While avoiding sports and/or school participation may be clear to most, how rest can be incorporated into all aspects of daily living may not be as apparent [9]. Results from recent research suggest that prolonged rest is associated with increased risk for development of secondary problems when normal activities are curtailed for extended periods of time [10–12]. These problems include, but are not limited to, physical deconditioning, anxiety/stress, social isolation, depression, irritability, and ‘acting out’ behavior at home and school [6, 13]. Of great concern is that many youth may be without early access to appropriate medical care and recovery guidance [14]. Lack of, or delayed access to care is problematic, as timely access to care may improve outcomes [14]. There is a need to provide guidance to youth and their families to optimize recovery and prevent the development of secondary problems following concussion.

Patient education [15] and self-management programs [4] provide guidance regarding concussion recovery. The effects of self-management programs on health outcomes are largely positive. In a meta-analysis of the effects of patient education on chronic health conditions, improvement in health was found in 64 % of 360 included studies [16]. Self-management programs, which typically include a patient education component, are reported to be effective for a range of chronic health conditions [17, 18]. A primary goal of self-management programs is to enable and empower patients to manage their own health [17, 18]. Through education platforms, patients identify and use strategies to manage their own unique health condition [17]. Promising results have been demonstrated in self-management programs in brain injury rehabilitation with adults [4, 19]. However, there are limited descriptions of these programs for concussion recovery in youth. Further, strategies developed for adult populations may not be suitable for youth populations.

Knowledge translation and exchange (KTE) principles can be used to support health education and self-management programs. In a review of knowledge transfer principles as applied to concussion in sport, Provvidenza and Johnston [5] concluded that knowledge and education can greatly influence the choices that athletes make concerning their health. Examples of the application of KTE in health education initiatives include utilizing audience-appropriate delivery methods to provide concise and consistent messaging, and involving the audience in the process [5, 20, 21].

The objectives of this study were to: 1) develop a self-management program curriculum for concussion recovery that is specifically targeted to youth and their families; 2) pilot test the program; and 3) evaluate program feasibility and knowledge uptake of participants.

## Methods

This program took place in a children’s rehabilitation hospital operating within a universal health care system in Toronto, Canada. This article describes the program development, implementation and evaluation phases. Ethics approval was received by the Research Ethics Board of Holland Bloorview Kids Rehabilitation Hospital and University of Toronto for retrospective secondary analysis of anonymous survey data previously collected for the purpose of program evaluation.

### Program development

The first phase was to develop a comprehensive evidence-informed concussion curriculum specifically designed for youth and their families. Curriculum development was based on synthesis of best evidence and expert opinion and was integrated within a self-management framework. In line with self-management and KTE principles specifically targeted to youth, the goals of “Concussion & You” are to: 1) provide evidence-informed, best practice guidance regarding concussion recovery; 2) enable participants to build their own concussion recovery ‘tool kit’; and 3) provide a platform for participants to interact and network with one another.

**Fig. 1:**
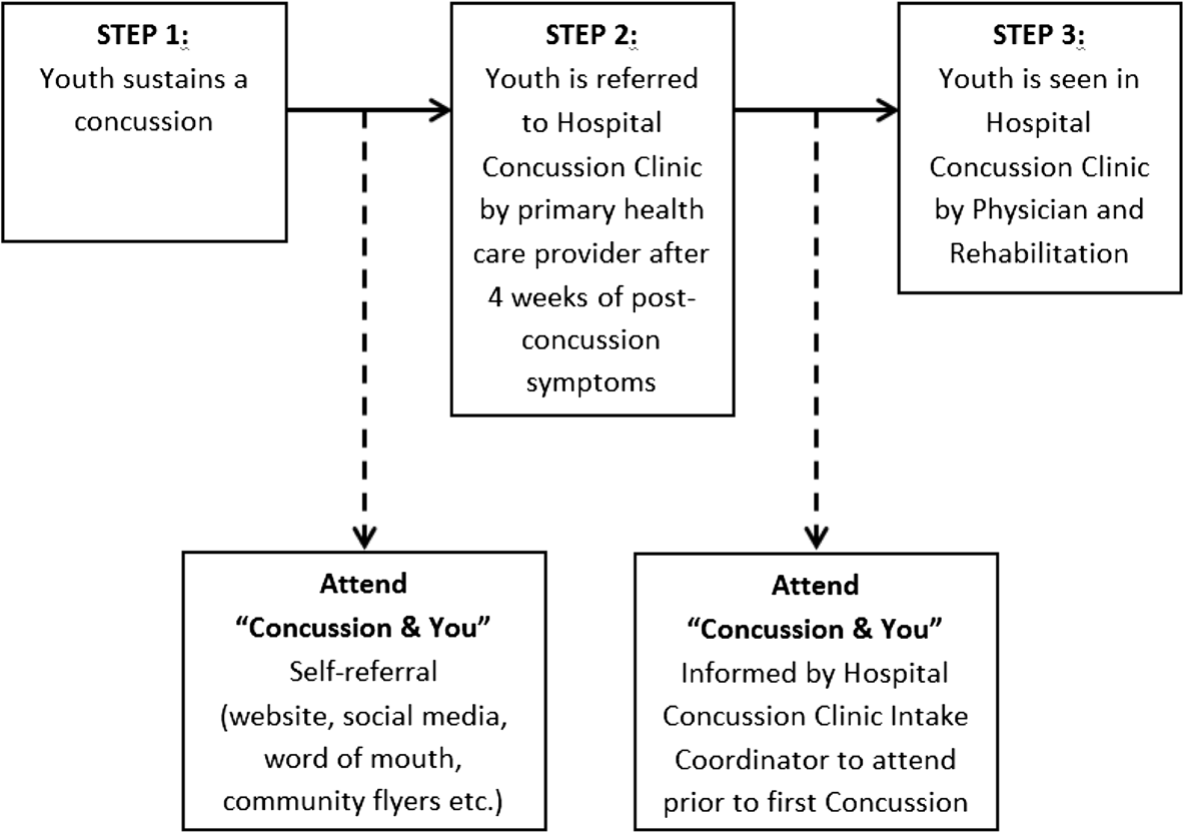
Access pathway to “Concussion & You concussion self-management program

Program development also considered the need to address existing gaps in service delivery. Figure 1 depicts how patients access the Hospital Concussion Clinic within the universal health care system model of care.

The program, “Concussion & You” was designed to serve both the hospital population and the general community at large. With respect to the hospital population, youth may be referred to the Hospital Concussion Clinic by their primary care provider if they have been experiencing post-concussive symptoms for longer than 4 weeks. Following referral, patients are contacted by the Hospital Concussion Clinic intake coordinator who advises them of their appointment and instructs them that the first part of their clinical care is to attend the “Concussion & You” session. Currently, there is a waiting period of approximately four to eight weeks from first point of contact with the intake coordinator until patients are seen by the concussion clinic physician. All patients and their families are directed to attend “Concussion & You” during this waiting period.

The program is also open to members of the general community who may attend the program via a self-referral process. “Concussion & You” is advertised through the hospital web page, social media (e.g. Twitter, Facebook), word of mouth, community outreach activities and posters. If needed, these youth may then seek referral to the Hospital Concussion Clinic from their primary care physician.

### Pilot testing: feasibility & knowledge uptake evaluation

The program was pilot tested from December 2013 to May 2015. A survey-based program evaluation design was used to evaluate feasibility and knowledge uptake. Prior to implementation, the survey was reviewed by concussion experts and a knowledge translation specialist for content and to ensure the survey used language that was easily understood by youth and parents. Surveys were administered at the education sessions and were collected anonymously. The complete survey can be accessed as a Additional file 1. The survey consisted of 11 questions, was designed to be short and easy to administer, and included both check box and open form questions. Survey questions were focused around the following:

1. Demographic data: There were two demographics questions. These questions were included to provide a better understanding of who was attending the sessions. The demographic data collected was limited to: if attendees were children/youth or parents/guardians; and, if attendees had received concussion education previously. As the original purpose of the surveys was to conduct program evaluation, identifiable information (e.g., name, address, date of birth etc.) could not be collected.
2. Feasibility data: Seven survey questions addressed feasibility (questions 1, 2, 7, 8, 9, 10, 11). These questions were included to give the researchers a better understanding of what participants felt was important about the program, and/or what was missing. Practical questions that may affect attendance were also asked.
3. Knowledge uptake data: Three survey questions targeted knowledge uptake (questions 4, 5, 6). These questions were aimed at identifying how participants’ understanding of concussion related topics changed from pre to post session. Using retrospective self-report, participants were asked to rate their pre- and post-session understanding of concussion recovery topics using a four point rating system (i.e., ‘very little’, ‘some’, ‘quite a bit’, or ‘a lot’ of understanding).

### Participants

Program participants were youth with concussion and their parents or caregiver, who voluntarily attended the program during the pilot phase and who agreed to complete the program evaluation survey.

### Data analysis

Descriptive statistics (e.g. frequencies) were used to retrospectively analyze surveys collected in the pilot test period. Data from questions involving a yes/no option or rating scale (closed questions) were analyzed using frequency counts/percentages. Data from open-ended questions were themed, whereby similar responses were grouped and recorded as a percentage of the overall responses.

## Results

### “Concussion & You” program description

“Concussion & You” is a ninety-minute interactive education session facilitated by occupational therapists and knowledge translation specialists with expertise in clinical concussion care and research. The program is designed for children and youth (ages 5-18) with concussion, and their families. A slide presentation and an accompanying written resource work booklet designed specifically for the program are used during the first hour of each session to guide attendees through the curriculum. While some information in this booklet was specifically written for “Concussion & You” (e.g., energy conservation curriculum), readily accessible best practice resources are also included (e.g., Canada’s Food Guide [22]; Return to Play Guidelines [23]). During the study period, the session was held two evenings per month. While the capability exists for web-based session participation for families who are unable to attend in person, evaluations in this study were only completed by participants who attended in person.

**Fig. 2:**
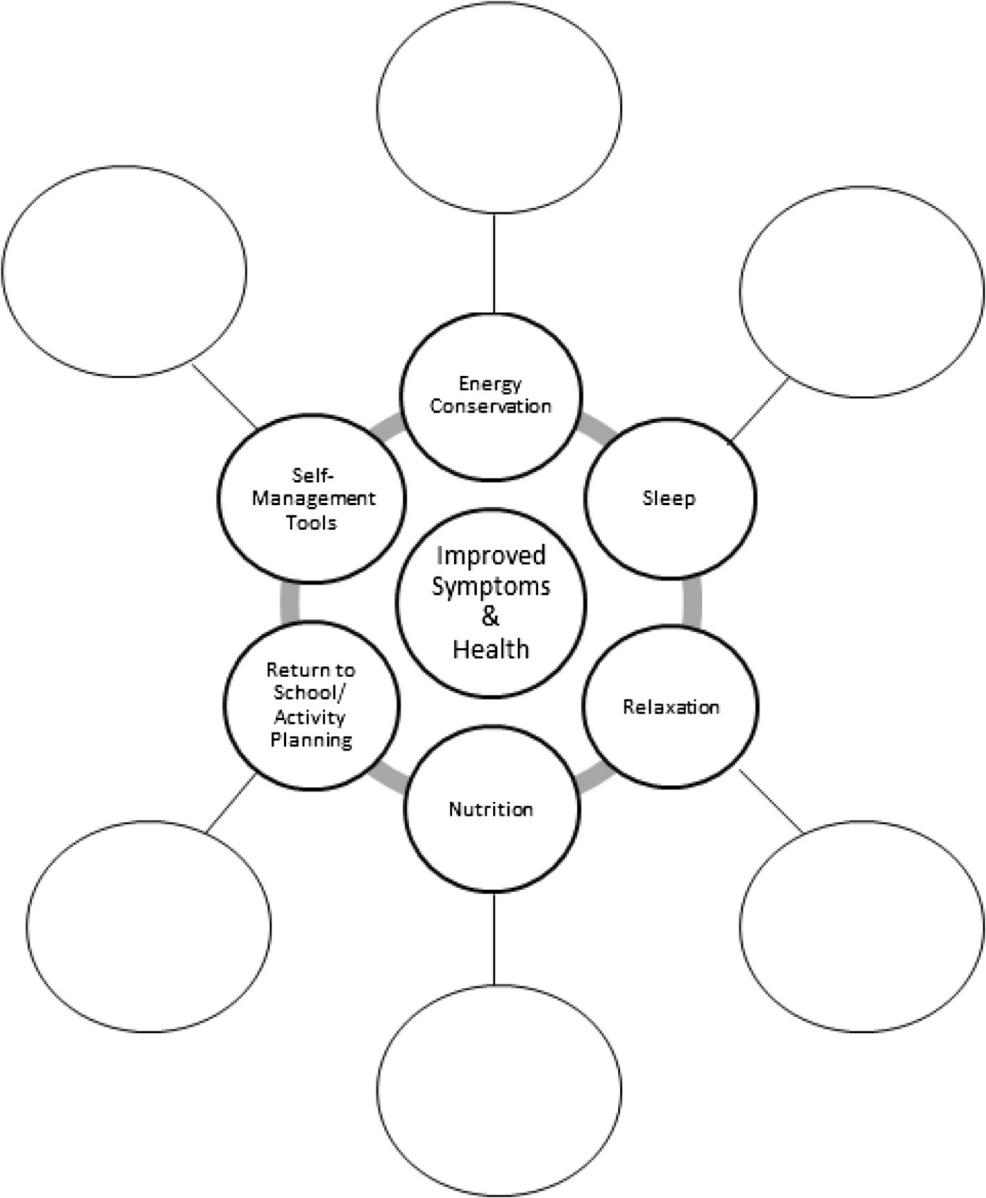
Visual Care Plan “Wheel of Health”

Each session begins with a general overview of concussion, including the definition and the range of symptoms experienced by youth with concussion. Participants are guided through strategies to explain their concussion to others (e.g., “ My concussion is like a broken bone but you just can’t see it. Like a broken bone, my brain needs to rest”). Analogies are offered to explain why youth might not be able to do their usual activities (e.g., “My body is like a car. It needs fuel/gas to run. Because of my concussion, it’s like my gas tank isn’t full. When my tank is low, it means I don’t have much energy. So I need to take breaks”). Using the gas tank analogy, participants are then guided through topics on a visual care plan or ‘wheel of health’ (Fig. 2).

Each of the six spokes on the wheel addresses a curriculum topic: 1) energy conservation; 2) sleep hygiene; 3) nutrition; 4) relaxation; 5) return to school, activities and sport; and 6) development of a concussion recovery ‘tool kit’. Participants are encouraged to fill in blank spaces on the wheel to build their concussion recovery tool kit; in other words, to identify strategies that they think might help to “keep their gas tank full”. Common risks for prolonged recovery and symptom exacerbation are discussed (e.g., returning to school too soon) and management strategies are identified. The last part of the session is aimed at answering questions, facilitating discussion and information sharing amongst program participants regarding their experiences.

### Demographics

A total of 87 surveys were completed by 62 parents and 25 youth who attended the program during the pilot phase. Half (52 %, 45/87) the participants reported no previous concussion education. Of the 48 % (42/87) who reported receiving previous concussion education, this education was primarily received from a health care professional (42 %) or the internet (36 %).

### Feasibility

An overwhelming majority (99 %, 86/87) reported enjoying the “Concussion &You” session and would recommend the program to others. Of the 60 respondents who indicated why they would recommend this program to others, 60 % (36/60) reported the program content. More specifically, participants reported that receiving comprehensive concussion management information from a credible source was very important. Both youth and parents gave an “excellent” rating for the following components: information presented (72 %, 63/87), delivery and format (72 %, 63/87), length of session (55 %, 49/87), ease of understanding (80 %, 70/87) and online session registration (68 %, 59-87).

### Knowledge uptake

**Fig. 3:**
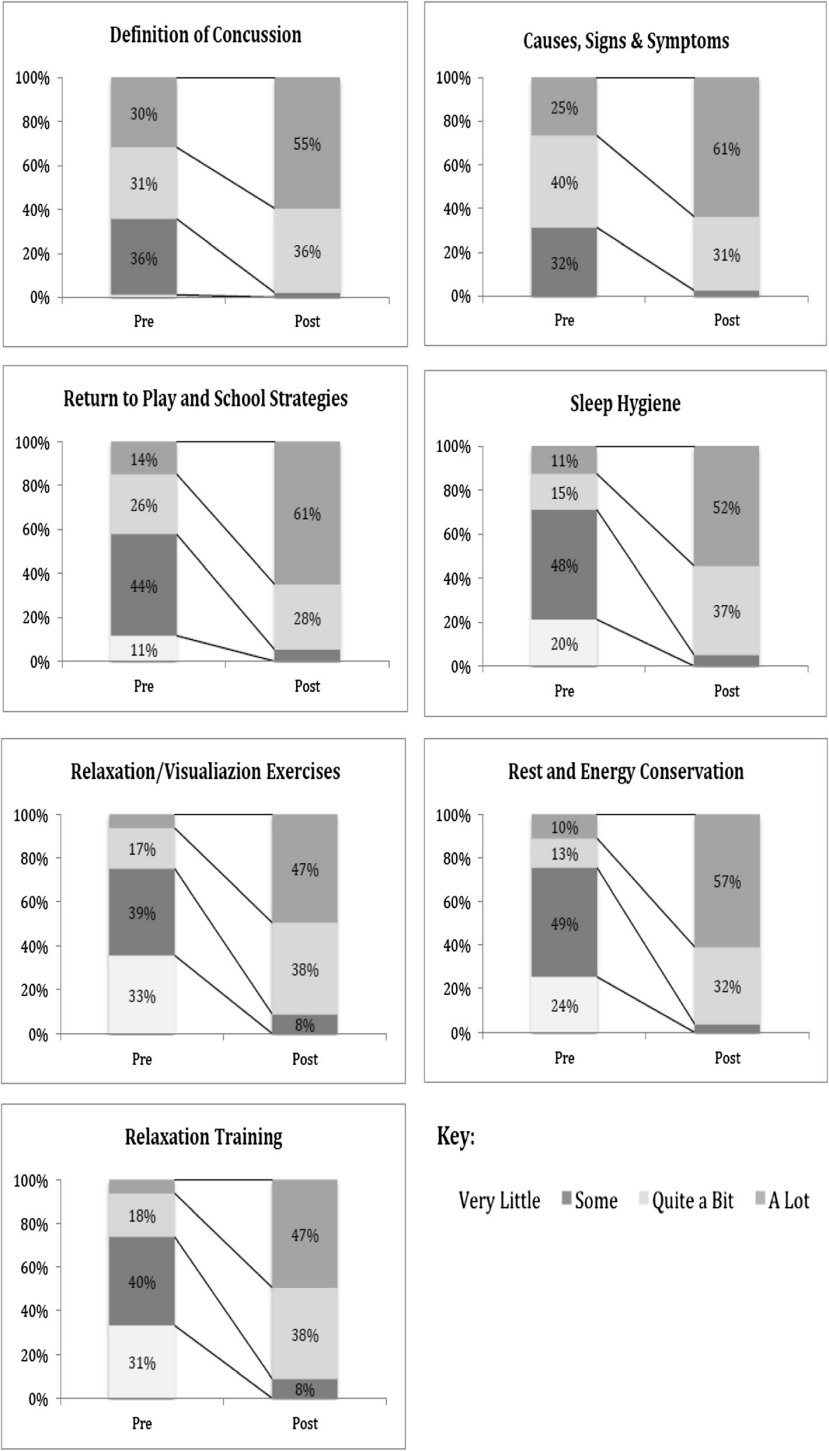
Pre-post self-reported changes in knowledge

Results from the knowledge uptake evaluation regarding session content are found in Fig. 3. Self-reported and retrospective increases in knowledge following the program are seen in all content areas.

Survey results indicated that most participants (94 %, 82/87) had intentions of incorporating their new knowledge into their daily routines and safety practices (100 % of parents and 80 % of youth). Of note, of the 50 respondents who identified how they plan to use this knowledge, 62 % (31/50) indicated that they would apply their knowledge to monitoring their child or themselves and 38 % (19/50) indicated that they planned to share program content with teachers and coaches.

## Discussion

“Concussion & You”, a self-management concussion education program was developed based on evidence-informed best practices and in alignment with principles of knowledge translation and exchange (KTE) [5]. The program was pilot tested for feasibility and knowledge uptake of participants using a program evaluation approach. Following a retrospective secondary analysis of the program evaluation surveys, results indicated that “Concussion & You” is a feasible means of delivering evidence-informed content to children and youth with concussion and their families. Principles of KTE help to explain the success of this program.

KTE means providing the right information to the right people in the right format at the right time and has been identified as a ‘missing link’ for enhancing concussion education [5]. “Concussion & You” meets the knowledge needs of users and directly addresses this missing link by: 1) making information accessible; 2) keeping messages simple and consistent; 3) promoting the sharing of experience; and 4) providing tangible tools and resources for participants.

### Making information accessible

To prevent development of secondary impairment and delayed recovery post-concussion, the provision of timely and relevant concussion education is paramount [5, 24]. “Concussion & You” aims to do this. Sessions are held frequently (bi-weekly) and are offered in the evenings to reduce conflict with school or work commitments. In the context of the Hospital Concussion Clinic Services, the “Concussion & You” program is delivered strategically to provide information to youth and their families during an identified gap in service delivery (i.e. waiting period). This program provides youth with concussion and their families timely access to evidence-based concussion education, support from other families and program leaders, and the opportunity to learn self-management strategies. “Concussion & You” supplements the services that can be offered within the limitations of the universal health system and related wait times.

Of the participants who indicated that they had received concussion education prior to attending “Concussion & You”, the most common information source was primary or emergency physicians. Participants reported that the information provided in “Concussion & You” would have been valuable to receive earlier in the recovery journey, to effectively manage concussion soon after injury.

### Keeping messages simple and consistent

As aligned with KTE principles and to promote uptake of information by knowledge users (e.g. youth and their families), delivering information in an understandable, consistent manner is an essential goal [5, 20, 21]. For example, within “Concussion & You”, the content delivered via the slide presentation was created specifically for youth. Individual slides use plain language by using smaller and fewer words, visuals and analogies to promote knowledge uptake by both youth and family members. The accompanying resource work booklet was designed to support and supplement the slide presentation content. Information is presented in the work booklet in the same order as the slide presentation to promote consistency in messaging. Finally, the messages and content delivered within the “Concussion & You” session match those delivered within the Hospital’s Concussion Clinic Service to promote consistency specific to the cohort of participants who may go on to receive additional rehabilitation within the Hospital Concussion Clinic.

### Promoting the sharing of experiences

Combining KTE [20] with the self-management program philosophy [25] of encouraging active engagement and participation, a portion of each “Concussion & You” session, is dedicated to providing youth and their families with a venue to discuss the impact concussion has had on their lives. Session leaders facilitate discussion between participants, asking those who are comfortable to share their experiences, and to exchange thoughts, ideas, strategies, and questions with the group. The full group (participants and session leaders) has the opportunity to respond to what is shared by individuals, providing a safe and collaborative environment to share knowledge and build relationships. To conclude “Concussion & You”, participants are encouraged to connect with each other, independent of the session leaders, to promote the creation of support networks that participants can access throughout their recovery.

### Providing tangible tools and resources

Common themes in self-management programmes include enabling and empowering patients to learn how to manage their health conditions [17, 18, 25]. “Concussion & You” is not designed to simply provide patient education, but rather to promote self-management and successful recovery *within* participants. Session content and the work booklet were designed to empower participants to create their own self-management concussion recovery ‘tool-kit’ that can be accessed throughout the post-concussion recovery process to optimally manage their injury. Daily planners, activity logs, and post-concussion symptom scales enable youth and their families to implement these strategies into their daily routines. This process is started in the session by providing participants with an opportunity to formally write out which strategies they will use on the ‘wheel of health’ (Fig. 2) to better manage their concussion. Participants are provided with the steps to take if their post-concussion symptoms persist and are given contact information for the concussion clinic intake coordinator along with other resources. Results from the “Concussion & You” post-session survey reflect that the strategy of using tangible tools does make a meaningful difference for participants in their recovery.

Other concussion education programs described in the literature (e.g. SLICE [26]) and accessible online (e.g. ‘Heads Up’ [27]) are primarily athlete-specific and focus on recognition of the signs and symptoms of concussion to enable athletes or coaches to remove themselves (or players) from games or practices and to seek assistance. General guidelines for recovery are offered in these programs. In contrast, “Concussion & You” is uniquely aimed at recovery from concussion and emphasizes use of specific self-management strategies to work toward return to activities including school, sport and social activities. Specific recovery tools are developed by each program participant that are unique for that individual.

### Limitations and future directions

The current study has limitations. Participants were drawn from individuals who were motivated to, and able to attend an early evening education program and were willing to complete a program evaluation survey following the program. Thus, the sample may not be representative of the general population of youth with concussion. While anecdotal and survey data suggest that participant response to “Concussion & You” has been overwhelmingly positive, a better understanding of the effects of this program on long-term knowledge gains and recovery is warranted. Empirical and qualitative inquiry to understand the effects of the program on recovery outcomes including symptom reduction, return to activity, prevention of secondary morbidity, and self-efficacy is needed. Identifying how this program contributes to recovery over time (i.e. follow up studies) is warranted. Further, although an important initial step in the evaluation and validation of this concussion education program, the retrospective secondary analysis of program evaluation surveys approach to this study resulted in the inability to collect more detailed demographic data (e.g., age, address/geographic location, time since injury etc.) that may be useful in determining the group specific impact of this program. Continued study that includes more rigorous research design is needed.

## Conclusion

“Concussion & You” is a patient education program that uses knowledge translation and exchange strategies to provide youth and their families with concussion recovery information to promote self-management of concussion. Results from our feasibility and knowledge uptake study indicate that this program is feasible, fills a health system service gap, is acceptable to youth and their families, and results in knowledge increases regarding concussion management.

## Additional file

Additional file 1: Program Survey: Concussion and You: Concussion education session for children and their families. (DOCX 70 kb)

## Abbreviation

KTE: Knowledge translation and exchange
