# Supplementary material for: Development and feasibility of an evidence-informed self-management education program in pediatric concussion rehabilitation
Source: BMC Health Serv Res. 2016 Aug 17;16:400. doi: 10.1186/s12913-016-1664-3 (PMC4989511; doi:10.1186/s12913-016-1664-3)
Supplement: Additional file 1: — Program Survey: Concussion and You: Concussion education session for children and their families. (DOCX 70 kb) [file 12913_2016_1664_MOESM1_ESM.docx]

***Concussion and You:***

***Concussion Education Session for Children and their Families***

| **Please indicate if you are a(n):**  **Youth**  **Parent**  **Other (please specify)________________** |
| --- |

The purpose of this brief survey is to determine the effectiveness and usefulness of the Concussion Education Session. Please take some time to complete the survey by answering the questions below. Ask the session facilitator to help you if you need it. Your feedback is important!

| 1. Did you enjoy the Concussion Education Session? (check one) | | |
| --- | --- | --- |
| a. |  | Yes |
| b. |  | No |

| 2. How would you rate the Concussion Session according to each of the following criteria: | | | | |
| --- | --- | --- | --- | --- |
|  | Poor | Fair | Good | Excellent |
| a. information presented |  |  |  |  |
| b. delivery and format |  |  |  |  |
| c. length of session |  |  |  |  |
| d. easy to understand |  |  |  |  |
| e. registration for session |  |  |  |  |
| f. facilities for session |  |  |  |  |

| 3. Have you been provided concussion education before? (check one) | | |
| --- | --- | --- |
| a. |  | Yes |
| b. |  | No |

**If you answered yes**, please explain the type of information you were provided and how the information was provided to you:

| 4. Through your training and/or past experience, which of the following topics, if any, did you **not** know about before attending this session? (check all that apply) | | |
| --- | --- | --- |
| a. |  | Definition of Concussion |
| b. |  | Causes, Signs & Symptoms |
| c. |  | Return to Play and School Strategies |
| d. |  | Sleep Hygiene |
| e. |  | Relaxation/Visualization Exercises |
| f. |  | Rest and Energy Conservation |
| g. |  | Relaxation Training |
| h. |  | Other (please list): ­­­­­­­­­­___________________________________________________ |
| i. |  | None |

| 5. Do you plan on making your knowledge of concussion part of your regular safety practice? (check one) | | | | | | | | | | | | | |
| --- | --- | --- | --- | --- | --- | --- | --- | --- | --- | --- | --- | --- | --- |
| a. | Yes | | c. | | Don’t know | | | | | | | | |
| b. | No | |  | |  | | | | | | | | |
| **If you answered yes,** please explain how you plan to use your concussion knowledge**. If you answered no,** please explain why you do not want to use your knowledge for safety practices.  Please Turn Over  6. Check the appropriate box to show your level of understanding of the listed topics **BEFORE** attending the session and **AFTER** attending the session. | | | | | | | | | | | | | |
| **Topics** | | **My understanding** | | | | | | | | | | | |
|  |  | **BEFORE Session** | | | | | | | **AFTER Session** | | | | |
|  |  | Very Little | | Some | | Quite a Bit | A Lot |  | | Very Little | Some | Quite a Bit | A Lot |
| a. Definition of Concussion | |  | |  | |  |  |  | |  |  |  |  |
| b. Causes, Signs & Symptoms | |  | |  | |  |  |  | |  |  |  |  |
| c. Return to Play and School Strategies | |  | |  | |  |  |  | |  |  |  |  |
| d. Sleep Hygiene | |  | |  | |  |  |  | |  |  |  |  |
| e. Relaxation/Visualization Exercises | |  | |  | |  |  |  | |  |  |  |  |
| f. Rest and Energy Conservation | |  | |  | |  |  |  | |  |  |  |  |
| g. Relaxation Training | |  | |  | |  |  |  | |  |  |  |  |

| 7. Would you recommend this session to others? (check one) | | |
| --- | --- | --- |
| a. |  | Yes b.  No |
| **If you answered yes,** why would you recommend this session? | | |
| **If you answered no**, why would you **NOT** recommend this session? | | |

| 8. As part of the session, you received a workbook/handouts. Are workbooks/handouts something that you value? (check one) |
| --- |
| a.  Yes b.  No |
| **If you answered no,** please explain why the workbook/handouts is not valuable and what other types of materials you would you like to receive as part of the session? |
|  |
|  |

| 9. What other ways would you like to receive concussion education? (e.g. DVD, printed materials, etc.) |
| --- |
|  |
|  |
| 10. Which topics should be removed from this session? What topics would you like to see addressed in future Concussion sessions? |
|  |
| 11. Please list any general comments and/or suggestions you may have. |

**Thank you for your feedback!**
